# Myricetin: targeting signaling networks in cancer and its implication in chemotherapy

**DOI:** 10.1186/s12935-022-02663-2

**Published:** 2022-07-28

**Authors:** Zeeshan Javed, Khushbukhat Khan, Jesús Herrera-Bravo, Sajid Naeem, Muhammad Javed Iqbal, Qamar Raza, Haleema Sadia, Shahid Raza, Munir Bhinder, Daniela Calina, Javad Sharifi-Rad, William C. Cho

**Affiliations:** 1 Office of Research Innovation and Commercialization, Lahore Garrison University, Lahore, Pakistan; 2 Department of Healthcare Biotechnology, Atta-ur-Rahman School of Applied Biosciences, National University of Sciences and Technology, Sector H-12, Islamabad, 44000 Pakistan; 3 Departamento de Ciencias Básicas, Facultad de Ciencias, Universidad Santo Tomas, Santiago, Chile; 4 Center of Molecular Biology and Pharmacogenetics, Scientific and Technological Bioresource Nucleus, Universidad de La Frontera, 4811230 Temuco, Chile; 5 Gansu Key Laboratory of Biomonitoring and Bioremediation for Environmental Pollution, School of Life Sciences, Lanzhou University, Lanzhou, 730000 China; 6 Department of Biotechnology, Faculty of Sciences, University of Sialkot, Sialkot, Pakistan; 7 Institute of Biochemistry and Biotechnology, University of Veterinary and Animal Sciences, Lahore, Punjab Pakistan; 8 Department of Biotechnology, Balochistan University of Information Technology, Engineering and Management Sciences, Quetta, 87100 Pakistan; 9 Department of Human Genetics & Molecular Biology, University of Health Sciences, Lahore, 54600 Pakistan; 10 Department of Clinical Pharmacy, University of Medicine and Pharmacy of Craiova, 200349 Craiova, Romania; 11 Facultad de Medicina, Universidad del Azuay, Cuenca, Ecuador; 12 Department of Clinical Oncology, Queen Elizabeth Hospital, Kowloon, Hong Kong China

**Keywords:** Myricetin, Cancer, Signaling networks, Apoptosis, Cell cycle, Nano-delivery

## Abstract

The gaps between the complex nature of cancer and therapeutics have been narrowed down due to extensive research in molecular oncology. Despite gathering massive insight into the mysteries of tumor heterogeneity and the molecular framework of tumor cells, therapy resistance and adverse side effects of current therapeutic remain the major challenge. This has shifted the attention towards therapeutics with less toxicity and high efficacy. Myricetin a natural flavonoid has been under the spotlight for its anti-cancer, anti-oxidant, and anti-inflammatory properties. The cutting-edge molecular techniques have shed light on the interplay between myricetin and dysregulated signaling cascades in cancer progression, invasion, and metastasis. However, there are limited data available regarding the nano-delivery platforms composed of myricetin in cancer. In this review, we have provided a comprehensive detail of myricetin-mediated regulation of different cellular pathways, its implications in cancer prevention, preclinical and clinical trials, and its current available nano-formulations for the treatment of various cancers.

## Introduction

The tumor micro-environment is diverse and involves complex molecular interactions ranging from cell-to-cell communication to metastasis and invasion [8, 35]. In cancer metastasis, cellular machinery is continuously under stress as a huge number of interfaces are taking place on a wide molecular landscape [65, 66]. A comprehensive understanding of the chain of molecular events taking place simultaneously in tumor cells helps in developing an efficacious drug and delivery system [94, 98]. The current therapeutic regimen for cancer has several drawbacks that make them aggressively toxic for both tumors as well as healthy cells [10].

Studies have found that naturally occurring phytochemicals have the therapeutic potential to treat human chronic diseases [9, 84, 100]. These phytochemicals target human long non-coding RNAs (lncRNAs) via RNAi technology. ROR, NEAT1, HI9, and PVTI are the commonest lncRNAs that are modulated by several phytochemicals including curcumin, sulforaphane, Epigallocatechin gallate (EGCG) and resveratrol. To enhance the efficacy of the treatment, conventional chemotherapeutic agents along with phytochemicals as a combination therapy can also be administered to patients [64, 69].

Myricetin, an isoflavonoid, is found in a wide variety of natural sources and is reported to contribute to alleviating numerous physiological anomalies such as cancers, cardiac diseases, inflammatory disorders, and neurological diseases. Several studies have highlighted the antioxidant and free radical scavenging properties of myricetin [6, 120]. Owing to these properties myricetin has been noted as a potential modulator of immune reactions and hypertension. Additionally, it has also been found to be an effective analgesic, anti-allergic and anti-inflammatory agent [2]. However, to perform the task, it makes molecular interaction with a wide range of proteins involved in cellular pathways related to cell survival, growth, differentiation, motility, homeostasis, and apoptosis. It is reported to alter the cell-to-cell molecular cascade in different human disorders [76, 86, 102, 116]. In healthy tissues, myricetin promotes signaling through the Akt pathway to induce cytoprotection, but in cancers, it suppresses this signaling cascade to induce apoptosis [40, 48]. In addition, it is also reported to promote TGFβ signaling in UV-exposed epidermal cells but TGFβ expression is down-regulated after myricetin treatment in the parasite-infected liver of mice [31, 71]. Thus, the current review aims to shed light on the interplay between myricetin and cellular pathways, its role in the prevention of cancer, current up-to-date knowledge regarding the nano-formulations of myricetin and selected clinical trials.

## Methodology

This review examines the modules and molecular mechanisms of action of myricetin and myricetin-mediated actions in cancer. For this study, databases were analyzed such as Web of Science, TRIP Database, PubMed/MedLine, Scopus and Google Scholar using the next MeSH terms**: “**Antineoplastic Agents/pharmacology”, “Apoptosis”, “Cell Line”, “Tumor”, “Cell Proliferation/drug effects”, “Down-Regulation”, “Flavonoids/pharmacology”, “Free Radicals”, “Myricetin”, “Neoplasms/drug therapy”, “Neoplasms/pathology”, “Reactive Oxygen Species”, “Signal Transduction/drug effects”. The most important mechanisms are represented in figures and tables. The chemical formula has been validated according to ChemSpider.

## Myricetin as anticancer agent: mediated targeting of cell signaling pathways and molecular implications

### PI3K/Akt and associated mTOR pathway

PI3K/Akt pathway in the cell is responsible for controlling various cell activities that are essential for its survival, growth, proliferation, and differentiation. It is shown through immunoblotting that myricetin activates PI3K/Akt signaling by promoting phosphorylation of Akt [40]. Its cytoprotective role is reported by numerous studies but in different cancers, myricetin interacts directly with Akt and suppresses its kinase activity. On a biochemical basis, it competes with adenosine triphosphate (ATP) to bind with Akt [48]. Additionally, myricetin is also reported to target PI3K expression in both in vitro and in vivo studies [37, 75]. Further, it also down-regulates epidermal growth factor receptor (EGFR) for targeting PI3K/Akt pathway [55].

Through targeting PI3K and Akt, myricetin also abrogates the activation of downstream molecular pathways in cancer. It interacts and prevents phosphorylation of both PI3K and Akt, leading to the disrupted mTOR activation [11, 42]. Furthermore, myricetin also modulates the expression of the mTOR pathway effector, p70s6k1 [122]. Regulation of PI3K/Akt/mTOR cascade via myricetin is illustrated to induce autophagy-mediated apoptosis in human colorectal carcinoma cells [128]. Furthermore, myricetin halts signal transduction through Akt/mTOR pathway by up-regulating the protein level of SIRT3 (Akt negative regulator). SIRT3 also suppresses Akt via the LKB1/AMPK pathway [79, 112].

### Raf/MEK/ERK pathway

Ras/Raf/MEK/ERK cascade is a significant signaling pathway of the biological system as it involves signals from a diverse range of molecular entities and the expression of genes associated with cell growth, progression, or death, depending on the sort of signal received [12]. Myricetin directly interacts with Raf/MEK/ERK and alters their kinase function. It binds with Raf in an ATP-noncompetitive manner and halts its activity to phosphorylate its downstream molecules [38]. Similarly, myricetin disrupts the kinase activity of MEK1 and suppresses phosphorylation of ERK, inducing an EGF stimulus [53]. It also reduces the protein levels of p38-MAPK as reported in various immunoblotting assays [127].

The myricetin-modulated Raf pathway also influences the associated signaling cascades and molecules in some diseases. For instance, in cancers, myricetin targets the expression of VEGF by directly inhibiting the activity of MEK1 and MKK4. In silico docking, the analysis revealed that myricetin binds with the ATP-binding site of MKK4 [44]. Likewise, it down-regulated MMP9 expression by inhibiting Raf [38]. FAK/ERK signaling cascade is also modulated by myricetin brings about the suppression of F-actin phosphorylation and down-regulates MMP2 and MMP9 [39]. Myricetin's potential to regulate MMPs is been exploited in cancer therapeutics, therapies related to inflammatory diseases, and anti-ageing research [13, 38, 47].

### NF-κB pathway

NF-κB signaling is also directly or indirectly modulated by myricetin. This pathway, particularly, participates in inflammatory responses and mediates the cell fate towards cell growth or death [59]. Myricetin-regulated Akt or MEK/ERK pathways are essential for inhibiting the activation of associated downstream NF-κB signaling. It halts the phosphorylation of Akt or ERK1/2 and resultantly, suppresses NF-κB [51, 101]. A study further elucidated through immunoblot assay that myricetin derivate, myricetin-3-O-b-D-lactose sodium salt, significantly reduces the protein levels of phosphorylated IKKα and IκBα [126].

Through suppressing the NF-κB pathway, myricetin also controls the signal transduction from BMP signaling. COX-2 activity is mediated by NF-κB. Myricetin by downregulating NF-κB activity also suppresses COX-2 expression [14]. PGE2 is activated by COX2, which promotes BMP expression. So, myricetin suppresses the BMP pathway via the COX-2/PGE2 cascade by inhibiting NF-kB.

### JAK/STAT pathway

JAK/STAT pathway has a central role in mediating cell inflammation and immune responses. It also employs NF-κB signaling to perform its designated function [90]. It is unveiled through pull-down assay that myricetin interacts with and inhibits JAK1, thus, consequently attenuating the phosphorylation-activation of STAT3. Myricetin treatment in JB6 cells inhibited cell transformation by down-regulating the JAK1-STAT3 pathway [49]. Suppression of STAT3 through myricetin action is also documented in cholangiocarcinoma cells [109]. STAT1 is specifically targeted by myricetin in ischemia-induced cardiac injury [88]. Another study revealed reduced phosphorylated-STAT3concentration, along with decreased HAMP mRNA levels, after myricetin treatment has alleviating role in iron-related toxicity in cells [15].

Its presence in a wide amount (8.80 mg/g) is determined in phenolic extracts of red vines through the Folin-Ciocalteu assay. In vitro analysis revealed that red vine extracts containing myricetin have an anti-inflammatory influence on colorectal HT-29 cells. These extracts mold phosphorylated-JAK1 protein levels and attenuate the nuclear localization of STAT3 [70].

### TNFα/TNFR signaling

TNFα signaling in a cell is primarily related to inflammation-induced cytotoxicity. Myricetin targets TNFα and disrupts the initiation of the TNFα/TNFR signaling cascade, resulting in down-regulation of downstream Akt, mTOR, and NF-κB pathways [126]. ERK also gets activated upon TNFα stimulus that contributes to cytotoxicity by activating JNK and NF-κB signaling [40, 101]. Hence, by targeting TNFα, myricetin abrogates signal transduction through all these pathways and contributes to cytoprotection. TNFα adaptor protein, TRAF6, is also modulated by myricetin. It induces ubiquitination of TRAF6 and inhibits downstream phosphorylation cascade TAK1/P38/JNK1/2 [58].

### Nrf2 pathway

Contrary to JAK-STAT, TNFα, and NF-κB signaling, the Nrf2 pathway acts to curb inflammation and cytotoxicity (induced by oxidative stress) [3]. The activity of Nrf2 is promoted while the NF-κB function is reciprocally suppressed by myricetin [58]. Myricetin modulates the Nrf2 pathway by preventing its ubiquitination-mediated destruction, leading to Nrf2 accumulation in the cytoplasm [78]. Accumulated Nrf2 translocation in a nucleus is also mediated by myricetin leads to activation of ARE which up-regulates ARE-associated genes’ expression [70, 78]. Derivative of myricetin also enhances the expression of Nrf2 associated gene, HO-1 which in turn, inhibits pro-inflammatory cytokines and induces expression of the anti-inflammatory cytokine, IL-10 [14].

### TGFβ/Smad pathway

TGFβ pathway has a major role in cell homeostasis and inflammation reduction. Signaling through TGFβ/Smad cascade is induced by myricetin [71]. Myricetin targets TFGβ1 expression to ameliorate noise-induced hearing loss [6]. Similarly, during pulmonary fibrosis, myricetin abrogates interaction between the cytoplasmic tail of the TGFβ receptor and HSP90β, leading to the inhibition of the Smad2/Smad5 complex [56]. A derivate of myricetin named as Myricetin-3-O-b-D-Lactose Sodium Salt also up-regulates the expression of TGFβ [126].

### BMP/SMAD and BMP-downstream pathways

BMPs belong to the superfamily of TGFβ protein. It is involved in the differentiation of mesenchymal stem cells into bone. Upon stimulus BMP receptors assemble a heterotetrameric form of SMADs and phosphorylate them, leading to the complex formation and their nuclear translocation. This signaling is also referred to as the canonical BMP pathway [114]. Myricetin is known to enhance protein levels of BMP1 that bolster signaling through both canonical (BMP-SMAD1/5/8) and non-canonical pathways (BMP/P38-MAPK) during osteoblast differentiation and maturation [28]. Further, myricetin also activates ERK, JNK, and P38-MAPK signaling cascades by enhancing the expression of BMP2 [43].

BMP pathway also plays a role in iron metabolism, by inducing hepcidin expression. Hepcidin acts as a modulator of iron metabolism and causes iron accumulation in cells, by down-regulating an iron transporter named ferroportin. Myricetin alleviates iron-associated cytotoxicity by suppressing gene expression of both ferroportin and hepcidin. Mechanistically, myricetin targets SMAD1 that in turn halts transcription of the HAMP gene [15].

### Wnt/β-catenin pathways

The Wnt pathway is involved in the regulation of cell fate determination, embryonic organogenesis, cell polarity, and cell migration [104]. Several studies indicate the targeting of the canonical wnt pathway by myricetin. It modulates the phosphorylation status of β-catenin and GSK-3β [43]. In breast carcinoma MCF-7 cells, myricetin promotes dephosphorylation of GSK-3β through inhibiting PAK1 protein expression. PAK1 attenuation abrogates the MEK/ERK signaling cascade, elevating the cytoplasmic concentration of unphosphorylated GSK-3β that captures β-catenin in the cytoplasm and subjects it to proteasomal degradation [36].

### Intrinsic apoptotic pathway

Pieces of evidence indicate that myricetin promotes apoptosis through different molecular pathways in different diseases. In resistant cancer cell lines, it enhances the activity of p53 and brings about cell death through both intrinsic and extrinsic pathways [30]. Furthermore, it also has the potential to trigger a caspase-independent cell death pathway. Kim et al. reported that myricetin promotes cell death by inducing Bax and AIF expression. AIF is further associated with the caspase-independent cell death pathway [7, 45].

Myricetin particularly promotes intrinsic apoptosis by directly promoting the expression and mitochondrial translocation of Bax and it is also reported to stimulate Bax-caspase3 signaling [36]. A study in hepatocellular carcinoma cell line HepG2 further explained the myricetin’s molecular mechanism in inducing intrinsic apoptosis. According to a study, myricetin promotes Bcl-2 down-regulation, it also induces Bax translocation to mitochondria and brings about cytochrome c release from the mitochondrial membrane. Myricetin triggers this process by attenuating Akt/ p70s6k1 signaling [122].

The apoptotic potential of myricetin is specific for affected cells. In healthy cells, it activates PI3K/Akt signaling and inhibits ERK/JNK pathway to induce cytoprotective influence. Mechanistically, it causes phosphorylation of Akt and prevents p38-MAPK phosphorylation. Through modulating these pathways, myricetin enhances Bcl-2 levels and prevents cytochrome c release from mitochondria, leading to the cell’s increased capability to tolerate and curb oxidative stress [40].

### Other pathways

Few studies have been conducted to delineate myricetin modulatory influence on the Hippo pathway. Its stimulus facilitates the Hippo pathway in its activation. It enhances the kinase function of LAT1/2 that phosphorylates YAP and subjects it to degradation [54]. Likewise, its role in the cell cycle is also reported. For instance, in cancer cell lines, it down-regulates cyclinD1 expression [36]. Similarly, attenuation of cyclinE1 is reported in endometriosis upon myricetin exposure [73]. However, these studies are scarce to understand myricetin's contribution to cell cycle regulation. This opens room for more investigation in determining myricetin's role in cell cycle regulation which could be extremely helpful in exploring its application in cancer treatment studies.

**Fig. 1:**
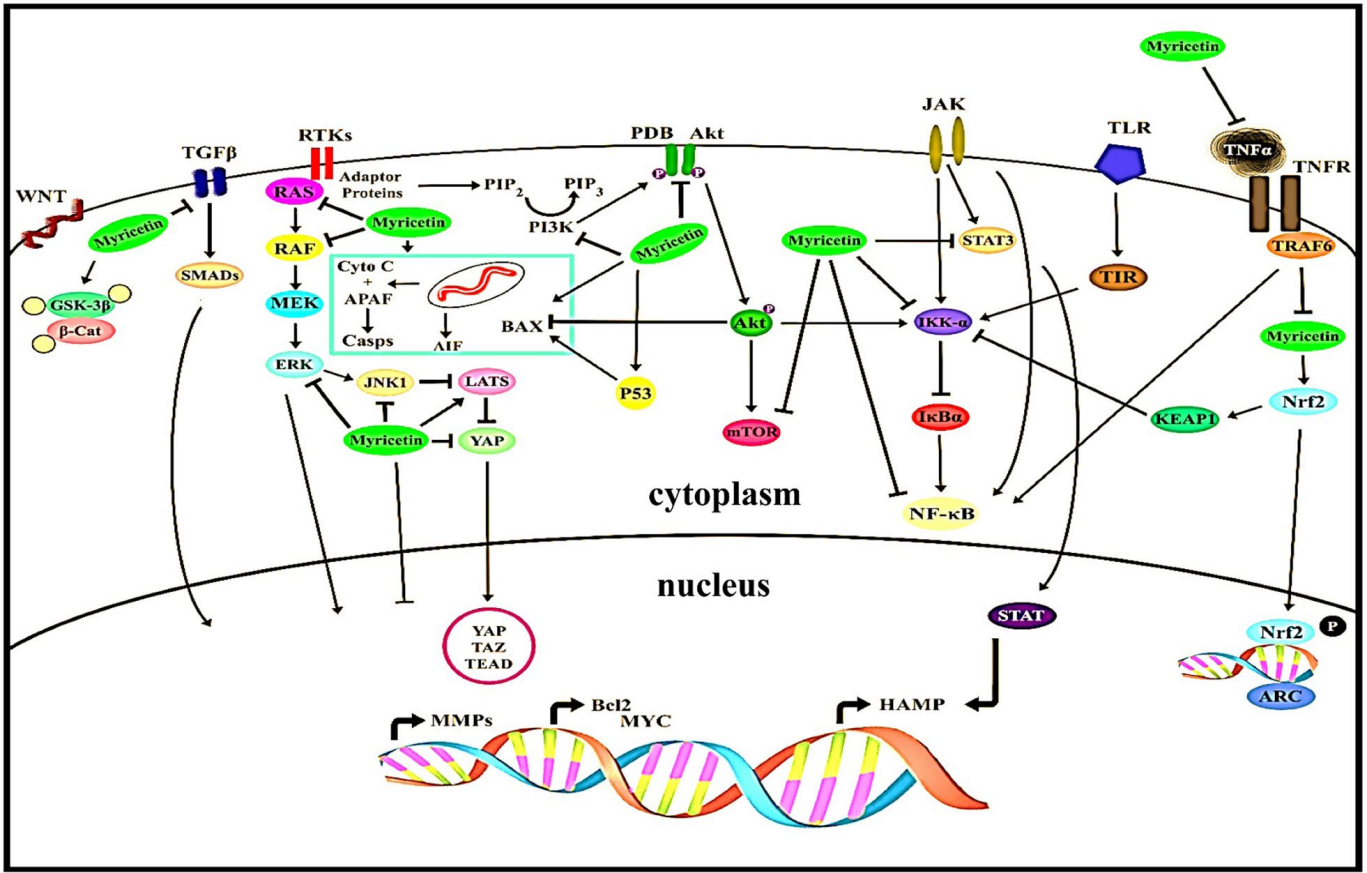
Myricetin regulatory influence on several cellular pathways. Myricetin modulates essential cellular pathways that support cell survival, growth, proliferation, cell cycle division, and apoptosis. PI3K/Akt and RAF/MEK/ERK signaling cascades are mainly influenced by myricetin action. Further, it acts as a negative modulator of the NFkB pathway, either by directly acting on it or by promoting signaling through the Nrf2 pathway. It also blocks JAK/STAT pathway. Myricetin also interacts with cell surface receptors of the RTKs family. It directly interacts with the TGF receptor and hinders TGF signaling. It also has a role in preventing beta-catenin accumulation in the cytoplasm by activating GSK 3b. Furthermore, it also turns on the hippo pathway by promoting LATS activity directly or through JNK. Myricetin's pro-apoptotic function is also known. It promotes Bax mitochondrial translocation through p53 activation or via Akt pathway inhibition

Figure 1 shows the myricetin regulatory influence on several cellular pathways.

## Myricetin as chemopreventive agent

It has been reported that myricetin is involved in the inhibition of cancer growth via regulating the expression of key enzymes involved in cancer progression and proliferation [60] (Table 1). A study conducted by Kim et al. reported that myricetin induces apoptosis in the HCT-15 cell lines of human colon cancer, in a dose-dependent manner. Myricetin promoted cytotoxicity in HCT-15 cells via activating the apoptotic genes such as the Bcl-2 associated X Protein. Dose-dependent (5-100 µM) presence of myricetin increased the Bax/Bcl-2 ratio ultimately leading to the recruitment of apoptotic machinery and consequently leading to cell death in HCT-15 cells [45]. Myricetin holds tremendous potential to be used as a potential therapeutic solution for colon cancer. Within molecular docking, cancer cell-based assays, and inhibitory mechanism studies it was found that myricetin prevents activation of human flap endonuclease 1 (hFEN1). The hFEN-1 is an endonuclease responsible for DNA replication and repair. The study showed that myricetin at a specific concentration (IC 50, 690 mM) prevents the attachment of the hFEN1 to Arg100 and Lys93 and consequently inhibits the proliferation of colon cancer [61].

**Table 1:** Established function of myricetin in different cancer cell lines

Cell lines/in vitro	Tumor type	Effect of Myricetin	Reference
DU145 PC3	Prostate Cancer	↓ metastasis, ↓epithelial-mesenchymal transition ↓invasiveness, ↑apoptosis	[118]
HCT-15 HT-29	Colon cancer	↑apoptosis ↓inhibits proliferation in a dose-dependent manner	[45, 52]
HepG2 A549 H1299	Hepatocellular Carcinoma	↑growth arrest, ↑ autophagy ↑growth arrest ↓proliferation	[54, 123, 124]
HGC-27 SGC7901	Gastric Cancer	↓ proliferation ↓growth ↑apoptosis in a dose-dependent manner	[20]
KYSE30 EC9706	Esophageal Carcinoma	↓proliferation, ↑apoptosis, ↓migration ↓invasion of tumor cells in a dose-dependent manner	[119]
MCF-7 MDA-Mb-231	Breast Cancer Cells	↑ apoptosis, ↓metastasis, ↓migration, ↓invasion	[36, 46]
OVCAR-3 A2780/CP70 SKOV3	Ovarian Cancer	↑ apoptosis ↓cells migration ↓angiogenesis	[125]
Panc1 S2-013 PaCa-2	Pancreatic Cancer	↑apoptosis	[75]
T24	Bladder Cancer	↑ growth arrest at M-phase, ↑apoptosis, ↓proliferation, ↓cell migration	[105]

Symbols: ↑ increase, ↓ decrease

Myricetin has also been reported to suppress angiogenesis in ovarian cancer cell lines. A study using in vitro and in vivo approaches showed that myricetin inhibited angiogenesis in a dose-dependent manner in OVCAR-3 cells [29]. The underlying mechanism involved in the suppression of angiogenesis is still unknown. Future investigations unveiling VEGF and other angiogenesis factors modulation through myricetin will enhance understanding of myricetin therapeutic potential.

In another ovarian cancer cell line SKOV-3, myricetin directly influenced cell viability in a dose-dependent manner. At a dose of 40 µg/ml, myricetin triggered apoptosis in SKOV-3 cells via activation of endoplasmic reticulum stress and DNA double-strand breaks [110]. Similar results were found in the study conducted by Zheng et al. in A2780 and OVCAR-3 cell lines of ovarian cancer [125]. From these findings, it can be justified that flavonoid myricetin has significant potential to be used as a potent inhibitor of ovarian cancer.

A recent study reported that myricetin can inhibit the proliferation and invasion of malignant cells in skin cancer. As potential anticancer mechanism, myricetin inhibited the activation of mitogen-activated protein kinase-1 (MEK1), which in turn prevented the activation of the downstream signal of the ERK/RSK AP-1 axis, thereby exerting inhibition of neoplastic transformation of skin cells. [41, 53]. Moreover, it has come to light less lately that myricetin can bind to central kinases such as the PI3K/Akt/JAK1, RAF1, MEK1, MKK4, and FYN. These kinases are involved in the regulation of a plethora of cellular processes and myricetin can directly inhibit such processes. It has also been investigated that myricetin inhibited the expression of tPA and EGF and explicitly inhibited growth in skin cancer cells in a dose-dependent manner [106]. Myricetin inhibits the growth of human lung adenocarcinoma cell line A549 cells in vitro. Myricetin directly targeted ERK signaling pathway and prevented the invasion and migration of A459 cells in a time-dependent manner [101]. It has also been demonstrated that a combination of myricetin and radiotherapy enhanced the sensitivity of tumor cells (A549 and H1299) toward radiation [121]. Another study has shown that a combination of myricetin and chemotherapy (5-fluorouracil) is efficient to prevent growth, invasion, and metastasis in the esophageal cancer cell EC9706 [113]. In addition to this, findings have also demonstrated the role of myricetin in the modulation and inhibition of bladder cancer. In human T24 bladder cancer cells, myricetin inhibited tumor growth and viability in a dose-dependent and time-dependent manner [105].

## Bioavailability

For centuries, plant extracts are used to treat almost all kinds of incurred illnesses, however, anciently, the source plants were solely chosen from past experiences or on a trial basis [89, 96, 99]. The plant extracts contained flavonoids and had been proved useful in treating various anomalies including cancers [72, 87]. In more recent times, the plant extracts are refined and detailed studies are carried out to further unearth the efficacy of these flavonoids and their specified use in treatment strategies[83, 85]. One such flavonoid, myricetin (3,5,7,3’,4’,5’-hexahydroxyflavone) has been found effective against several diseases including cancers. It is readily found in numerous edible parts of plants, including herbs and teas as well as fruits such as oranges, grapes, and berries. Myricetin is a lipophilic and weak acidic compound that works best at pH 2.0 and with low aqueous solubility (16.60 g/mL), making it insoluble in the gastrointestinal tract and thus limiting its efficacy via oral absorption [102, 117].

Despite clinical advancements in human health and cancer treatment, optimal gut absorption and solubility are the two stumbling blocks that have significantly hampered the drug efficacy in treating cancer. The drug concentration and mode of delivery greatly affect its efficacy, thus various strategies have been proposed [81, 95].

Recent developments have attempted to enhance myricetin bioavailability by designing a drug delivery system based on nanotechnology [115]. Such efforts have been made previously on several natural compounds whose therapeutic efficacy was hampered by poor aqueous solubility [33, 34].

## Ready-to-go strategies to increase bioavailability and anticancer efficacy: nanoformulations of myricetin

To overcome the low bioavailability and increase oral delivery of myricetin, alternative approaches such as nanoformulation of myricetin are done to improve solubility, drug delivery, and efficacy. In this section, we will discuss different strategies for nanoformulation of myricetin. Many studies have achieved significant improvements to curb various cancers by using myricetin nanoformulations.

**Fig. 2:**
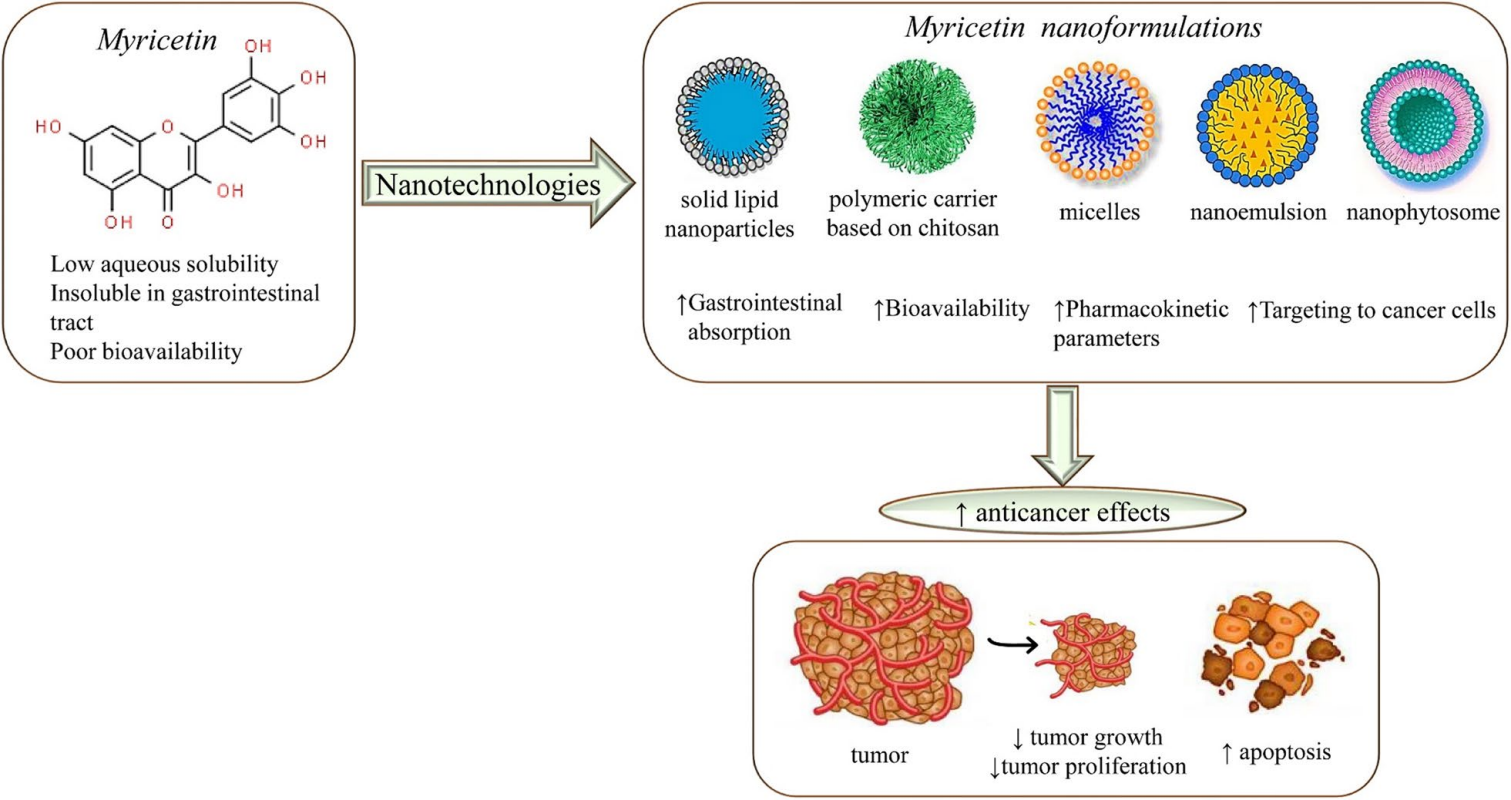
Beneficial nanomedicines of myricetin against cancer. Symbols: ↑increase, ↓decrease

Nanoformulation is carried out in numerous ways owing to a wide range of shapes and forms of nanoscale particles [19, 63]. Nanoformulations including nanocrystals, nanoemulsions, polymeric nanoparticles, dendrimers, carbon nanotubes, polymeric micelles, and lipid nanocarriers, are exploited for their ability to circumvent the poor oral bioavailability of insoluble drugs [74]. So various studies have exploited certain advantages of each type and have made a wide range of myricetin-nanoformulations available for use in cancer and other treatments (Fig. 2). The nanoformulation could also provide an added advantage of targeted delivery of drugs. It is achieved by furnishing formulations to target a specific molecule, thus reducing the risk of wastage of dose at one side and protecting other sites from the toxicity and side effects of the drug. Moreover, high efficacy is achieved with low dosage [82]. The synthesis of metal nanoparticles encapsulating drugs including flavonoids is well established and tested practice.

In a recent study, the development of nano-emulsifying drug delivery systems (SNEDDS) of myricetin was achieved, which was formulated in various phases of excipients, constructing pseudo-ternary phase diagrams, and optimizing based on droplet size and emulsification efficacy after drug loading [77].

Another strategy is the microemulsion formulation to improve the issues of drugs. A microemulsion formulation was successfully developed by Guo et al. resulted in efficiently delivering and increased antiproliferative activity by myricetin in human cancer HepG2 cells without damaging normal cells of surroundings. This formulation consisted of Cremophor RH40 (12%), Tween 80 (6%), Transcutol HP (9%), WL 1349 (18%) and distilled water (55%). This microemulsion significantly increased the solubility of myricetin 1225 times more than myricetin alone [24].

Myricetin micelles (Myr-MCs) have also been formulated using a range of micelles compounds for their utilization in the treatment of various anomalies. One such nanocarrier was HS15-Myr micelle based on polyoxyl 15 hydroxystearate micelles encapsulating myricetin. The resulting ultra-small-sized micelles (12.17 ± 0.73 nm) demonstrated higher aqueous stability, storage temperature range, and membrane permeation than the free myricetin solution among the accepted pH ranges for eyedrops. These were also seen to permeate into the cornea of a mouse without any damage and showed anti-inflammatory properties [27].

A recent study has synthesized myricetin-mediated silver nanoparticles (Myr-AgNPs) using a green approach to aim for improved therapeutic efficacy of myricetin. They found that the resulted NPs were spherical and showed promising antibacterial effects [57]. In another example, spherical myricetin-gold nanoparticles (Myr-AuNPs) with sizes less than 50 nm were synthesized by adopting a simple and stable ultrasound-assisted method. Interestingly, the graph-theoretical network analysis revealed mTOR as an effective target for Myr-AuNPs in breast cancer cells which was further confirmed by in silico molecular docking. Further studies to assess the anti-cancerous effect of Myr-AuNPs revealed that it could decrease cell viability by acting as a pro-apoptotic agent and could also depolarize mitochondrial membrane potential and elevate reactive oxygen species [67].

Gaber et al. encapsulated myricetin into Gelucire-based solid lipid nanoparticles (SLNs). They proposed that fat-soluble antioxidant in SLN helps the drug survive at a higher temperature. Moreover, cell media is usually not suitable for the survival of the flavonoid, so physiological buffers, as well as simulated fluids, should be supplemented with stabilizers additives. With all their recommendations, they found 300-fold lesser drug degradation rates and 4500-fold increased half-life of myricetin [21], thus clearly indicating the importance of nanoformulation of myricetin and other flavonoids.

The development of inhalable myricetin solid lipid nanoparticles (SLNs) for lung cancer therapy has produced encouraging results. The formulation could be done in two steps, producing complexation of myricetin with phospholipid Lipoid-S100 and nanoencapsulation in Gelucire-based, surfactant-free SLNs, which could result in 75.98 nm diameter nanoparticles with a zeta-potential of -22.5 mV, and encapsulation efficiency of 84.5%. Subsequent experiments with these inhalable myricetin solid lipid nanoparticles showed enhanced cellular uptake and increased efficacy of myricetin with a threefold reduction in IC_50_ values [68].

Myricetin-loaded NLCs (nanostructured lipid carriers) have also been utilized in combination with another drug. For example, myricetin-loaded NLCs in combination with docetaxel (DXT) in MDA-MBA231 breast cancer cells increased the percentage of apoptosis. The expression of anti-apoptotic genes, Cyclin B1 and Mcl1 was reduced, while the expression of pro-apoptotic factors such as Bax and Bid was significantly increased. [62].

To increase the low solubility of myricetin, Hong et al. synthesized nanosuspensions with myricetin, the particle size ranging from 300 to 500 nm. The results of the study showed that myricetin nanosuspensions had a higher solubility in vitro and efficacy compared to simple myricetin powder [25].

Folic acid (FA)-conjugated bovine serum albumin (BSA) nanoparticles (NPs) encapsulating myricetin could be targeted to bind to folate receptor (FR) positive breast cancer cells. The Myr-loaded BSA NPs were further assembled by a modified desolvation cross-linking technique. An FA-conjugated carrier, N-hydroxysuccinimide (NHS)-FA ester, was successfully synthesized by Kunjiappan et al. This resulted in NPS non-covalently bound to folate receptors and demonstrated fast release of myricetin in targeted breast cancer cells where they appeared to cause a significant decrease in cell viability of targeted cells [50]. In another study, myricetin-loaded mesoporous silica nanoparticles (MSN) combined with multidrug resistance protein (MRP-1) siRNA showed excellent target accuracy in non-small cell lung cancer (NSCLC) cells. Again, FA-conjugation directed the nanoformulations to target lung cancer cells exclusively and the release of the drug into the cells resulted in a decrease in cell viability in myr-nanoformulations treatment combined with MRP 1 than myricetin alone in A549 and NCI-H1299 cell lines [103].

The bioavailability of myricetin can also be increased by the formulation of nanophytosomes. A group of researchers developed nanophytosomes with myricetin using phosphatidylcholine. Myricetin nanophytosomes were formulated using a thin-layer hydration-sonication method with a varied ratio of myricetin, phosphatidylcholine, and cholesterol, respectively. The results of the study showed that the formulation myricetin: phosphatidylcholine: cholesterol in the ratio 1: 1: 0.4 has the highest bioavailability. [17].

Formulation of polymeric carrier based on chitosan-functionalized Pluronic P123/F68 micelles encapsulating myricetin has also shown promising results against glioblastoma cancer. These myricetin micelles (Myr-MCs) exhibited improved cellular uptake and antitumor activity compared to free myricetin in vitro and in vivo. These micelles could also affect apoptotic proteins such as Bcl-2, Bad and Bax in mice [111].

## Clinical perspectives on anticancer potential of myricetin

### Recent clinical studies

Myricetin in combination with different phytochemicals has been clinically tested for the treatment of diabetes. In a clinical trial spanning over 4 weeks, Blueberin and myricetin were administrated in a quantity of 250 mg blueberry leaves and 50 mg myricetin (300 mg together) on daily basis. This placebo-controlled trial revealed that blueberin in combination with myricetin successfully reduced plasma sugar levels in type-2 diabetic patients. The use of blueberin reduced plasma sugar levels from 143 ± 5.2 mg/L to 104 ± 5.7 mg/L. Blueberin was also found to reduce the serum levels of alanine aminotransferase (ALT), AST, and glutamyltransferase (GGT) and also minimized the levels of C-reactive protein (CRP) which could ultimately prevent inflammation [1]. A blend of chlorogenic acid, myricetin, and quercetin is also known as Emulin has been reported to decrease blood glucose levels in type-2 diabetes patients. The clinical trial of Emulin was conducted on 40 subjects having a range of BSF (blood sugar fasting) between 126 and 249 mg/mL [4]. In addition to its role in diabetes, myricetin has been reported to be tested for its chemopreventive abilities and as an inhibitor of cell proliferation and signaling [16]. In one of the surveys, it was found that the use of myricetin as a dietary supplement prevented the symptoms of prostate cancer and reduced risk [23]. In another clinical study, intake of myricetin in combination with other flavonoids such as apigenin, kaempherol, quercetin, and luteolin prevented the incidence of ovarian cancer [22] however, no exact cause of disease incidence was correlated with any of the above flavonoids [16, 22]. Myricetin as a dietary supplement has also been reported to decrease the risk of the development of lung cancer. In a meta-analysis, it was demonstrated that myricetin and other flavonoids could prevent the risk of lung cancer by 30–40% in 5073 lung cancer patients that were examined [108].

### Therapeutic limitations and clinical gaps of myricetin as anticancer agent

Traditional medicinal herbs have majorly contributed to the pharmaceutical industry. But these products possess challenges for drug discovery and drug optimization. In this current era, these challenges are addressed by scientific and technological developments including advancements in microbial culturing, genome mining, and enhanced efficacy of analytical tools. Due to this, the emerging chemotherapeutic resistance challenge can be addressed [5]. According to the studies, reactive oxygen species (ROS) majorly contribute to the initiation, proliferation, metastasis, and anti-apoptotic behavior of cancer cells. Several cells signaling pathways are responsible for the oncogenic behavior of ROS [92, 97]. Radiotherapy, chemotherapy, and surgery are the only effective techniques to combat advanced stages of cancer approximately till the age of 50 years [18] Traditional herbs and their structural analogues are the alternatives and biocompatible approaches to combat cancer cell proliferation and metastasis, thus, directly affecting the prognosis and overall survival rate in cancer patients [26, 32]. There has been a recent surge in the field of phytochemistry that promoted the exploration of a broad range of phytochemicals with the potential to reduce the toxicity caused by the current drugs used for the treatment of cancer [80, 91, 93, 107].

Myricetin has plenty of health benefits and despite its evident role in the treatment of cancer, very limited data is available that justifies its thorough clinical implications. However, emerging preclinical evidence has begun to shed light on the therapeutic efficacy of myricetin. Although emerging preclinical evidence has begun to shed light on its potential anticancer efficacy, there are numerous limitations which need further studies in the future.

An important therapeutic limitation is the lack of translational studies to establish the routes of administration, the exact effective doses for each type of cancer, or combinations with other cytostatic drugs. Another clinical limitation is due to the poor pharmacokinetic characteristics and low potency, as a result, nanoformulations with myricetin to be transported directly to targeted cancer cells were the most promising strategy for increasing its bioavailability. Also, stronger analogues of myricetin may be developed to increase its anticancer potency.

More research is needed to understand in-depth the potential anticancer mechanisms of myricetin, and it is unclear whether these effects are the result of a direct effect on malignant cells or as a result of binding to extracellular targets or effects on angiogenesis.

## Overall conclusion

Cancer is a multifactorial disease and several cellular pathways responsible for cell growth, survival and proliferation are modulated simultaneously in disease development and progression. Effective drugs for cancer must have the potential to target different molecular players belonging to different cell signaling cascades. Myricetin is an isoflavonoid that is usually present as glycoside in different fruits, vegetables, nuts, berries and herbs. It has also been found as an important constituent of wine, tea, and some medicinal plants. Several cellular pathways modulated by myricetin in humans are discussed in detail in this paper. Myricetin has been demonstrated to modulate cell pathways essential for supporting tumor cell survival such as PI3K/Akt pathway, nrf signaling, canonical and non-canonical wnt pathway, mTOR pathway, Ras/Raf pathway and JAK/STAT pathway. The interplay between myricetin and signaling pathways sheds light on its importance as a therapeutic solution for cancer. However, further exploration is still required to sketch out the most probable targeting of the cellular network. The major stumbling block to using myricetin at the clinical level is its poor water solubility, pH and low bioavailability. Plenty of research is in the phase of pre-clinical trials to establish nano-formulations of myricetin that can enhance its bioavailability and absorption but still requires herculean efforts to bring it to clinical trials and human use. Considering the importance of myricetin as an important modulator in-depth analysis of its interaction with non-coding RNAs will bring up a new avenue in the smart targeting of cancer cells. Considering such benefits putting myricetin to use as a therapeutic agent has been challenging because of its limited bioavailability and solubility. Nano formulation at in vitro and in vivo levels has proven its significance in enhancing the bioavailability and treatment efficacy of myricetin. The incorporation of myricetin into nanoformulations can be a promising therapeutic option. Further research delineating the dosage safety of myricetin nanoformulation will be a step forward in its clinical application.

## Data Availability

Not Applicable.

## Abbreviations

lncRNAs: Human long non-coding RNAs
RNAi: RNAi
NEAT1: Nuclear paraspeckle assembly transcript 1
EGCG: Epigallocatechin gallate
PI3K: Phosphatidylinositol-3-kinase
mTOR: Mammalian target of rapamycin
ATP: Adenosine triphosphate
EGFR: Epidermal growth factor receptor
MEK: Mitogen-activated protein kinase kinase
ERK: Extracellular signal-regulated kinase
MKK4: Mitogen-activated Protein Kinase Kinase 4
NF-κB: Nuclear factor kappa-light-chain-enhancer of activated B cells
IKKα: Inhibitory Kappa B Kinase α
IκBα: Nuclear factor of kappa light polypeptide gene enhancer in B-cells inhibitor, alpha
BMP: Bone morphogenetic protein
COX-2: Cyclooxygenase-2
PGE2: Prostaglandin E2
JAK/STAT: Janus kinase-signal transducer and activator of transcription
STAT: Signal transducer and activator of transcription
HAMP: Human hepcidin gene
TNF: Tumor necrosis factor
TRAF6: Tumor necrosis factor receptor (TNFR)-associated factor 6
TAK1: Transforming growth factor-β-activated kinase 1
Nrf2: Nuclear factor erythroid 2-related factor 2
TGF-β: Transforming growth factor-β
SMAD: Suppressor of Mothers against Decapentaplegic
GSK3β: Glycogen synthase kinase
Bax: B-cell Leukaemia/Lymphoma 2-associated X Protein
AIF: Apoptosis-inducing factor
Bcl-2: B-cell lymphoma 2
LAT1: L-Type Amino Acid Transporter
TGF: Transforming Growth Factor
JNK: Jun N-terminal Kinase.
hFEN1: Human flap endonuclease 1
VEGF: Vascular endothelial growth factor
DNA: Deoxyribonucleic Acid
RSK: Ribosomal protein S6 kinase
tPA: Tissue plasminogen activator
EGF: Epidermal growth factor
ERK: Extracellular signal-regulated kinase
Myr-AuNPs: Spherical myricetin-gold nanoparticles
SLNs: Solid lipid nanoparticles
NLCs: Nanostructured lipid carriers
MCL1: Myeloid Cell Leukemia 1
